# Comparative analysis of phyto-fabricated chitosan, copper oxide, and chitosan-based CuO nanoparticles: antibacterial potential against *Acinetobacter baumannii* isolates and anticancer activity against HepG2 cell lines

**DOI:** 10.3389/fmicb.2023.1188743

**Published:** 2023-05-31

**Authors:** Muhammad Hassan Sarfraz, Muhammad Zubair, Bilal Aslam, Asma Ashraf, Muhammad Hussnain Siddique, Sumreen Hayat, Jorrdy Neves Cruz, Saima Muzammil, Mohsin Khurshid, Muhammad Farrukh Sarfraz, Abeer Hashem, Turki M. Dawoud, Graciela Dolores Avila-Quezada, Elsayed Fathi Abd_Allah

**Affiliations:** ^1^Institute of Microbiology, Government College University, Faisalabad, Pakistan; ^2^Department of Bioinformatics and Biotechnology, Government College University, Faisalabad, Pakistan; ^3^Department of Zoology, Government College University, Faisalabad, Pakistan; ^4^Laboratory of Functional and Structural Biology, Institute of Biological Sciences, Federal University of Pará, Belém, Brazil; ^5^Department of Physics, COMSATS University Islamabad, Lahore, Pakistan; ^6^Botany and Microbiology Department, College of Science, King Saud University, Riyadh, Saudi Arabia; ^7^Facultad de Ciencias Agrotecnológicas, Universidad Autónoma de Chihuahua, Chihuahua, Mexico; ^8^Plant Production Department, College of Food and Agricultural Sciences, King Saud University, Riyadh, Saudi Arabia

**Keywords:** *Acinetobacter baumannii*, chitosan, copper oxide, nanoparticle, antimicrobial activity, anticancer activity

## Abstract

The aim of this study was to provide a comparative analysis of chitosan (CH), copper oxide (CuO), and chitosan-based copper oxide (CH-CuO) nanoparticles for their application in the healthcare sector. The nanoparticles were synthesized by a green approach using the extract of *Trianthema portulacastrum*. The synthesized nanoparticles were characterized using different techniques, such as the synthesis of the particles, which was confirmed by UV–visible spectrometry that showed absorbance at 300 nm, 255 nm, and 275 nm for the CH, CuO, and CH-CuO nanoparticles, respectively. The spherical morphology of the nanoparticles and the presence of active functional groups was validated by SEM, TEM, and FTIR analysis. The crystalline nature of the particles was verified by XRD spectrum, and the average crystallite sizes of 33.54 nm, 20.13 nm, and 24.14 nm were obtained, respectively. The characterized nanoparticles were evaluated for their *in vitro* antibacterial and antibiofilm potential against *Acinetobacter baumannii* isolates, where potent activities were exhibited by the nanoparticles. The bioassay for antioxidant activity also confirmed DPPH scavenging activity for all the nanoparticles. This study also evaluated anticancer activities of the CH, CuO, and CH-CuO nanoparticles against HepG2 cell lines, where maximum inhibitions of 54, 75, and 84% were recorded, respectively. The anticancer activity was also confirmed by phase contrast microscopy, where the treated cells exhibited deformed morphologies. This study demonstrates the potential of the CH-CuO nanoparticle as an effective antibacterial agent, having with its antibiofilm activity, and in cancer treatment.

## 1. Introduction

Developing antimicrobial resistance is one of the major health-related issues worldwide (Morris and Cerceo, 2020). Since the discovery of penicillin, antimicrobial drugs have revolutionized the healthcare system and have also contributed to developing drug resistance in microorganisms (Tacconelli et al., 2018). Resistance in microbes is continuously evolving with time, and it is proving difficult for the existing drugs to keep pace with the emerging pathogens. Moreover, with the increasing resistance, there has also been a decline in the development of new antibiotics along with their continuous overuse and misuse, which is further complicating the issue. Furthermore, the surge in multi-drug-resistant (MDR) bacteria has also worsened the situation, as it is proving difficult for antibiotics to treat infections caused by such bacteria (Medina and Pieper, 2016). *Acinetobacter baumannii*, a Gram-negative, coccobacillus, non-fastidious, and nosocomial pathogen, is gaining attention due to its resistance against a spectrum of antibiotics such as polymyxins and carbapenems. It is rapidly emerging in hospital-acquired infections, where it is majorly involved in persistent infections such as bacteremia, pneumonia, urinary tract infections, meningitis, endocarditis, and wound infection, and therefore, it is considered a paradigm of MDR bacteria (Vázquez-López et al., 2020). The Center for Disease Control (CDC) has listed highly virulent bacteria (ESKAPE pathogens) that can cause multiple nosocomial infections and show resistance against a wide range of antibiotics, among which *Acinetobacter baumannii* was named (Kyriakidis et al., 2021). Owing to the serious health concerns, the World Health Organization listed *A. baumannii* among 12 MDR bacterial families that pose a threat to the health sector, and thus it has called for the development of alternative strategies to deal with such resistant infections (Mat Rahim et al., 2021).

Similarly, cancer is also one of the most complicated and challenging issues, and various research across the world is being conducted to find an effective cure with least side-effects. Currently, early-stage cancers are treated with radiotherapy, chemotherapy, and surgery. These treatments, although effective to an extent, hold the risk for various side-effects and thus are more costly for patients (Qian et al., 2017). Therefore, there is a need to explore inexpensive alternatives with the potential for both the diagnosis and treatment of cancers and resistant infections, where emerging technologies can be utilized to design the required tools and effective drugs with minimum possible risks for society.

This century has witnessed a huge role of nanotechnology in revolutionizing all realms of sciences. There has been a giant leap in the search for and synthesis of nanoparticles in which multiple roles in various multi-disciplinary research areas have been discovered. The increasing popularity of nanoparticles owes to their beneficial properties, in contrast to bulk materials, i.e., high surface area, sensitivity, porosity, reactivity, catalytic activity, and biocompatibility, and thus, they have been extensively studied for their various impacts on living cells (Oluwasanu et al., 2019). Nanotechnology provides the opportunity to make use of the significant attributes of metal oxides in nanoparticles by expanding their potential in the drug delivery system, cell labeling, environmental remediation, and antimicrobial agents, as biomarkers and nanodrugs for diseases such as cancer and diabetes. Various metal oxide nanoparticles such as silver oxide (Ag2O), magnesium oxide (MgO), copper oxide (CuO), iron oxide (FeO), zinc oxide (ZnO), and selenium oxide (SeO_2_) have been synthesized and studied for their multiple applications (El Shafey, 2020).

CuO nanoparticles stand prominently due to their biological, chemical, and physical attributes. The supreme biocompatibility of CuO, along with its high stability, photovoltaic activity, non-toxicity, antibacterial potential, anticancer activity, thermal conductivity, lower cost, longer lead time, and antioxidant activity, makes it a favorable candidate for multi-purpose applications (Padil and Černík, 2013; Akintelu et al., 2020; Sackey et al., 2020). Investigators all over the world have authenticated the significant roles of CuO nanoparticles for their antimicrobial and antibiofilm applications against drug- resistant pathogens along with various biological efficacies (Farag et al., 2020; Shehabeldine et al., 2021; Doghish et al., 2022; Okba et al., 2022; Shehabeldine et al., 2023). The potential properties of these metal oxide nanoparticles can be further enhanced by complexing them with organic or inorganic biopolymers. The induction of the biopolymer not only enhances the properties of the nanoparticles but also provides them with additional beneficial aspects (Sarkar et al., 2012). Chitosan, a natural polymer present in the shell of sea crustaceans, has been used with preference for the synthesis of nanoparticles owing to its bioavailability, biodegradability, non-toxicity, biocompatibility, and environmental safety (Sánchez-Machado et al., 2019; Singh et al., 2021). Additionally, chitosan also possesses antimicrobial and antioxidant properties, which can act synergistically with the CuO nanoparticles to expand the antimicrobial spectrum. Numerous studies have employed chitosan for the synthesis of different metal oxide nanoparticles and authenticated wide range applications for these nanoparticles in numerous fields (Sunil, 2013).

The current investigation explores the potential of the CH, CuO, and CH-CuO nanoparticles for their antibacterial and anticancer potential. The study employed the biological synthesis of the nanoparticles by using the aqueous extract of *Trianthema portulacastrum*. The nanoparticles were characterized for their synthesis by different techniques and then employed for to study their antibacterial and antibiofilm potential against *Acinetobacter baumannii* isolates and anticancer activity against HepG2 cell lines.

## 2. Materials and methods

### 2.1. Bacterial and plant samples

Two drug-resistant strains of *Acinetobacter baumannii* (accession no. MH605335 and MH605336) were obtained from the Department of Microbiology, Government College University Faisalabad (GCUF), Pakistan. For the nanoparticle preparation, fresh leaves of *Trianthema portulacastrum* were obtained and verified from the Department of Botany, GCUF. The chemicals used in this study were obtained from Sigma-Aldrich (St. Louis, MO, United States).

### 2.2. Preparation of plant extract

For the biosynthesis of the nanoparticles, aqueous leaf extract was initially prepared (Nayak et al., 2015). The fresh leaves of *Trianthema portulacastrum* were taken and washed with double-distilled water to remove any impurities. The washed leaves were air dried and converted to powder form using a mixer grinder. Leaves in powdered form were then subjected to Soxhlet extraction using deionized water for 72 h. The obtained aqueous extract of the plant was then filtered using Whatman filter paper no.1, and the filtrate of the plant extract was collected.

### 2.3. Biosynthesis of nanoparticles

The CH-CuO nanoparticles were synthesized following a previously reported protocol (Maheo, 2022). A total of 50 mL of 0.05 M copper sulphate solution was mixed with 20 mL of leaf extract via continuous stirring for an hour. After stirring, the pH of the solution was then adjusted to 9 by adding 0.2 M NaOH dropwise. Meanwhile, chitosan solution was prepared by dissolving 20 mg of chitosan in 20 mL acetic acid solution (1.5%) under stirring at 60°C. A total of 20 mL of chitosan solution was then added to the plant extract–copper sulphate mixture, and the solution was then stirred continuously for 2 h at 60°C. The solution was centrifuged at 10,000 rpm for 20 min, and the nanoparticles at the bottom were filtered, washed with double-distilled water, and dried. For the biosynthesis of the CuO and chitosan (CH) nanoparticles, a similar protocol was followed, except for the addition of chitosan and copper sulphate solutions, respectively.

### 2.4. Characterization of CH-CuO nanoparticle

The characterizations for the synthesized nanoparticles were caried out at the Life Sciences Division Center, Zhejiang University, China. The surface morphology, shape, and size of the nanoparticles were determined by scanning electron microscopy (SEM; SU-8010, Tokyo, Japan) and transmission electron microscopy (TEM; JEM-1230, Akishima, Japan) analysis. The synthesis of nanoparticles was confirmed by recording the UV–visible spectra of the nanoparticles using a UV spectrophotometer in the range of 200 nm–800 nm. Fourier transmission infrared spectroscopy (FTIR; Bruker, Germany) was used to confirm the presence of different functional groups, which was confirmed by recording the peaks in the range of 500–4,000 cm^−1^. The crystallinity of the nanoparticles was authenticated by employing x-ray diffraction analysis (STOE-Germany). The diffraction pattern was measured using Cu Kα rays at 2θ range from 10° to 80°. The crystallite size of the nanoparticles was calculated using the Debye–Scherrer equation.

### 2.5. Antibacterial activity

Agar well diffusion was performed to determine the antibacterial potential of the prepared nanoparticles against *Acinetobacter baumannii* isolates (Venkataraju et al., 2014). The bacterial isolates were refreshed by incubating the culture in Luria–Bertani (LB) broth for 24 h at 37°C. After incubation, the turbidity of the cultures was adjusted to 0.5 McFarland standard. Muller Hinton agar plates were prepared, and 0.1 mL of freshly prepared bacterial culture was poured and swabbed over the plates. The plates were allowed to dry, and wells were punched in the plates using a sterile well cutter. Meanwhile, colloidal solutions of the CH, CuO, and CH-CuO nanoparticles (1 mg/mL) were separately formed in dimethyl sulfoxide (DMSO; 0.1%), which were then sonicated at 30°C for 30 min. A total of 50 μL of each nanoparticle solution, taken from their respective stocks, was separately dispensed in the wells of the plates. The plates were incubated for 24 h at 37°C. Following incubation, the zones of inhibition were measured. Ceftazidime was used as the positive control for the experiment.

The MIC (minimum inhibitory concentration) for the nanoparticles against the bacterial isolates was estimated by a broth microdilution assay (Dutra-Correa et al., 2018). A 96-well microtiter plate was used, in which two-fold serial dilution of the nanoparticles (0–1,000 μg/mL) was performed using Mueller–Hinton broth. The freshly prepared bacterial culture (24 h old) was taken, and turbidity was adjusted to 0.5 McFarland standard. A total of 100 μL of the bacterial culture was added to each well containing different concentrations of the nanoparticles, and the microtiter plate was incubated for 24 h at 37°C. Following incubation, NBT dye (nitro-blue tetrazolium chloride) was added to each well. The viability of the bacterial cells was determined by observing the change in color from yellow to blue. The lowest concentration of the nanoparticles that prevented the color change was termed MIC.

For the MBC (minimum bactericidal concentration) determination of the CH, CuO, and CH-CuO nanoparticles, 24 h-old *Acinetobacter baumannii* cultures with the turbidities adjusted to 0.5 McFarland standard were used. The bacterial culture was incubated (24 h; 37°C) in Mueller–Hinton broth test tubes with different concentrations of the nanoparticle equal to MIC and above. Following incubation, freshly prepared MH agar plates were used, where 100 μL of the culture from each tube was spread over the plates, which were then incubated at 37°C for 24 h. After incubation, the plates were observed for bacterial growth. The minimum concentration at which no bacterial growth was seen was termed MBC.

### 2.6. Antibiofilm potential of the nanoparticles

The crystal violet method was employed to determine the antibiofilm efficacy of the CH, CuO, and CH-CuO nanoparticles against *Acinetobacter baumannii* isolates (Rivera Aguayo et al., 2020). Bacterial culture was grown overnight, and its turbidity was adjusted to 0.5 McFarland standard. A microtitration plate assay was performed where 180 μL of MHB was dispensed in the wells, followed by the addition of 10 μL of different nanoparticle concentrations (0.25x MIC, 0.5x MIC, and 1x MIC). A total of 10 μL of the bacterial culture was added to the wells containing different concentrations of nanoparticles, and the plate was incubated for 24 h at 37°C. Following incubation, the free-floating cells were discarded, and the cells attached at the bottom of the wells were fixed via methanol fixation. The biofilm cells were stained by crystal violet stain (0.1%) for 10 min. The stained cells were washed with deionized water and suspended in glacial acetic acid (33% v/v). The optical density was measured through an ELISA reader at 570 nm. The control was followed through similar processes, except for the addition of the nanoparticles. The percent inhibition of the biofilm was calculated as follows:


$$\begin{array}{l} \text{Percentage inhibition}=1-\text{Absorbance of cells treated with}\;\text{NPs}/\\ \text{ Absorbance of untreated cells}\times 100 \end{array}$$

### 2.7. Antioxidant activity

The antioxidant activity of the nanoparticles was determined via a DPPH assay (Jain et al., 2020). A total of 10 μL of each nanoparticle was separately added to 180 μL of DPPH solution (0.1 mM). The mixtures were stirred and incubated in the dark for 30 min at 37°C. Following incubation, absorbance was measured at 517 nm. A change in color from deep violet to yellow indicated a radical reduction. Ascorbic acid was used as a control for the experiment. The percent antioxidant activity was evaluated as follows:


$$\text{Antioxidant activity}\;\left(\%\right)=\left[\left({\text{OD}}_{C}\_{\text{OD}}_{S}\right)/\left({\text{OD}}_{C}\right)\right]\times 100$$

where OD _C_ represents absorbance for the control, while OD_S_ represents absorbance for the sample.

### 2.8. Cytotoxicity analysis

Cytotoxicity analysis was performed to establish the non-toxic dose of the nanoparticles. Vero cell lines, derived from the normal kidney cells, were obtained from the cell culture lab at the Department of Bioinformatics and Biotechnology, GCUF. An MTT [3-(4,5-dimethylthiazol-2-yl)-2,5-diphenyltetrazolium bromide] assay was used to evaluate the cytotoxicity doses of the nanoparticles (Revathi and Thambidurai, 2019). In a 96- well microtiter plate, 100 µl of cells (1 × 105 cells/mL) were added into the wells containing 200 µl Dulbecco’s Modified Eagle’s Medium (DMEM) and 10% fetal bovine serum (FBS). The plate was incubated at 37 °C under 5% CO2 and 90% humidity for 24 h. Meanwhile, nanoparticle solutions at different concentrations (50 µg/ml, 100 µg/ml, and 150 µg/ml, 200 µg/ml) were prepared in 0.1% DMSO. Then, 100 µl of nanoparticle solution from each concentration was separately added to the wells containing the cells, and the plate was incubated at 37 °C under 5% CO2 and 90% humidity for 48 h. The cControl was processed without the addition of nanoparticles. After incubation, 15 µl of MTT (5mg/ml in PBS) was added to the wells, and the plate was incubated for a further 4 h at 37 °C. A total of 100 µl DMSO was added following incubation to dissolve the formed formazan crystals. The absorbance of the cells was measured at 570 nm using an ELISA plate reader. The mean of the cell viability values was compared to the control to determine the effect of the nanoparticles on the cells. The cell viability (%) was plotted against the concentration of the nanoparticles.

### 2.9. Anticancer activity

Human hepatocellular carcinoma HePG2 cells were obtained from the cell culture laboratory at the Department of Bioinformatics and Biotechnology, GCUF. HepG2 cell lines (passage no. 4) having with a concentration of 1 × 10^5^ cells/mL were used for the experiment. The anticancer activity of the nanoparticles was evaluated using an MTT [3-(4,5-dimethylthiazol-2-yl)-2,5-diphenyltetrazolium bromide] assay. A total of 100 μL of cells were added into the wells of the microtiter plate containing DMEM (200 μL) and 10% FBS. The plate was incubated at 37°C under 5% CO_2_ and 90% humidity for 24 h. Then, 100 μL of nanoparticle solution from each concentration, i.e., 25 μg/mL, 50 μg/mL, and 100 μg/mL, was separately added to the wells containing the cells, and the plate was incubated at 37°C for 48 h. The assay was performed in triplicates for each dilution of nanoparticles, and the control was processed without the addition of nanoparticles. Following incubations, an MTT assay was performed as described above, and the absorbance was measured at 570 nm. The viability of the cells was evaluated as follows:


$$\begin{array}{l} \text{Cell inhibition}\;\left(\%\right)=100-{\text{Abs}}_{570}\;\text{of treated cells}/\\ \;{\text{Abs}}_{570}\;\text{of control cells}\times 100 \end{array}$$

### 2.10. Phase-contrast microscopy of HepG2 cells

The morphological changes in the cell lines were examined using phase-contrast microscopy, as reported previously (Syed Abdul Rahman et al., 2013). HepG2 cells (1 × 10^5^) were added in the DMEM medium containing 10% FBS in tissue culture dishes. Then, 1 mL of different concentrations (12.5 μg/mL, 25 μg/mL, and 50 μg/mL) of nanoparticles were added to the medium, and the culture dishes were incubated for 24 h at 37°C under the required conditions of CO_2_ (5%) and humidity (90%). Following incubation, the media in the plates were discarded, and PBS was used to wash the HepG2 cells. The control cells were processed similarly, except for the addition of the nanoparticles. For observing the morphological changes, digital imaging via a charge-coupled device (CCD) SP 480 H color camera (Olympus, Japan) was performed.

### 2.11. Statistical analysis

The experiments in this study were run in triplicates, for which the results were presented as mean ± standard deviation. ANOVA and Student’s *t*-test were performed, and the obtained results were considered statistically significant at *p* < 0.05.

## 3. Results

### 3.1. Characterization

**Figure 1:**
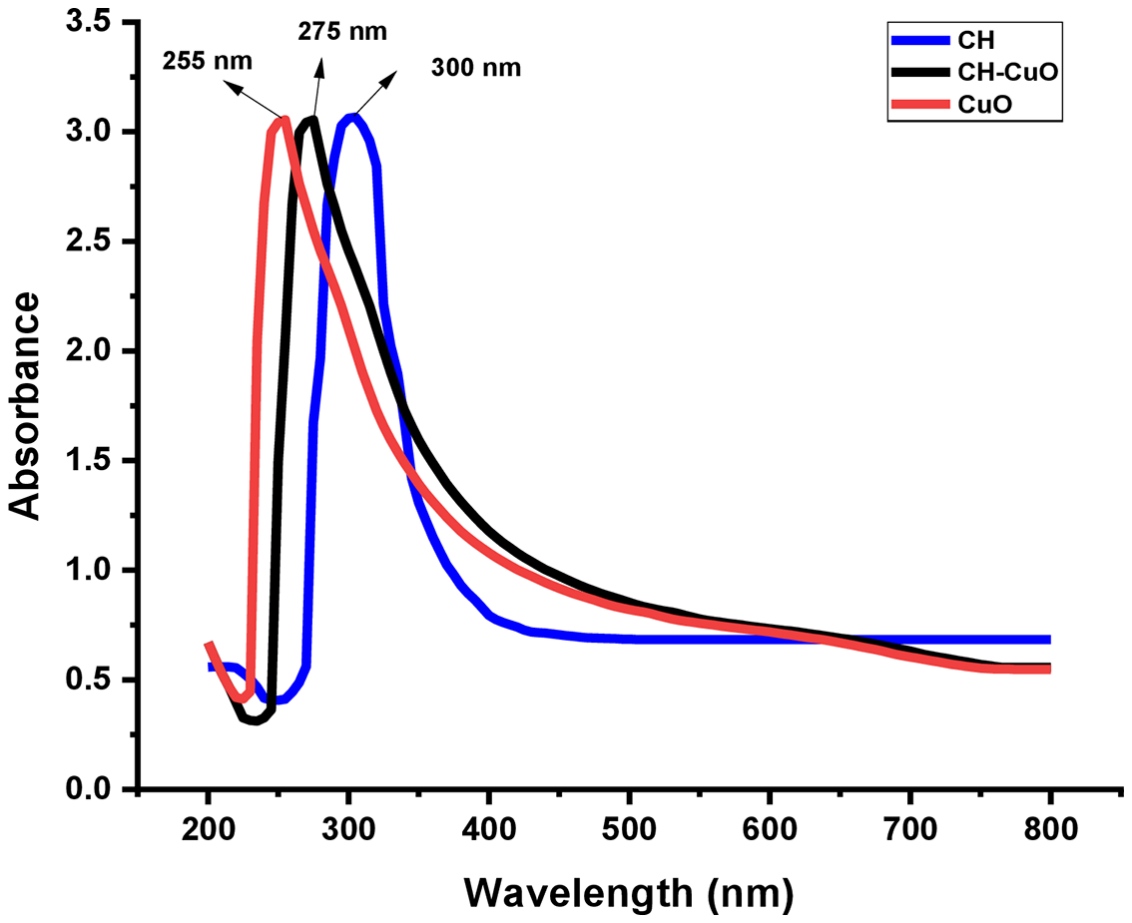
UV–visible spectra for green-synthesized CH, CuO, and CH-CuO nanoparticles showing absorption peaks at 300 nm, 255 nm, and 275 nm, respectively.

**Figure 2:**
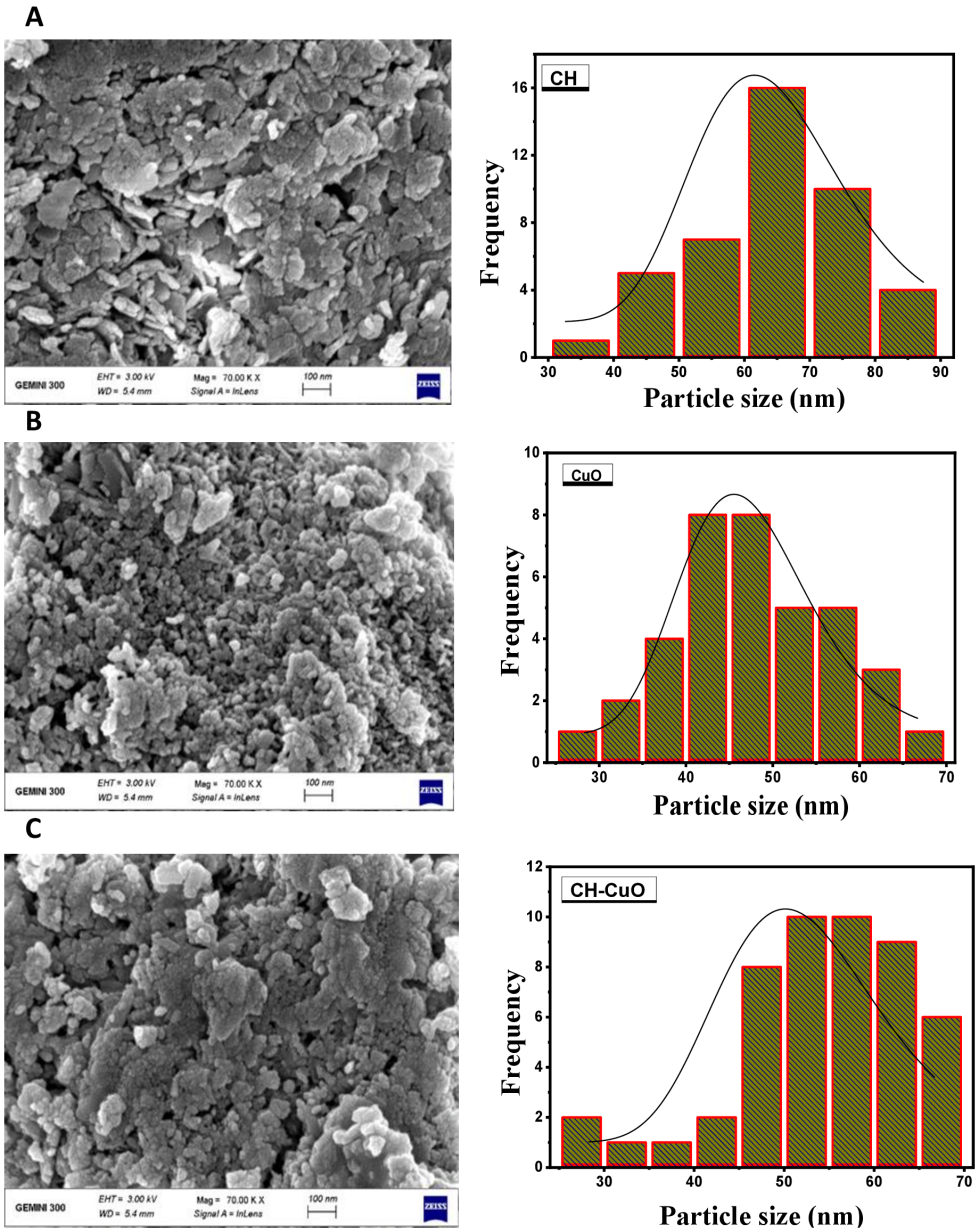
SEM images for the **(A)** CH, **(B)** CuO, and **(C)** CH-CuO nanoparticles. Graphs showing particle size for the nanoparticles are presented along the images.

**Figure 3:**
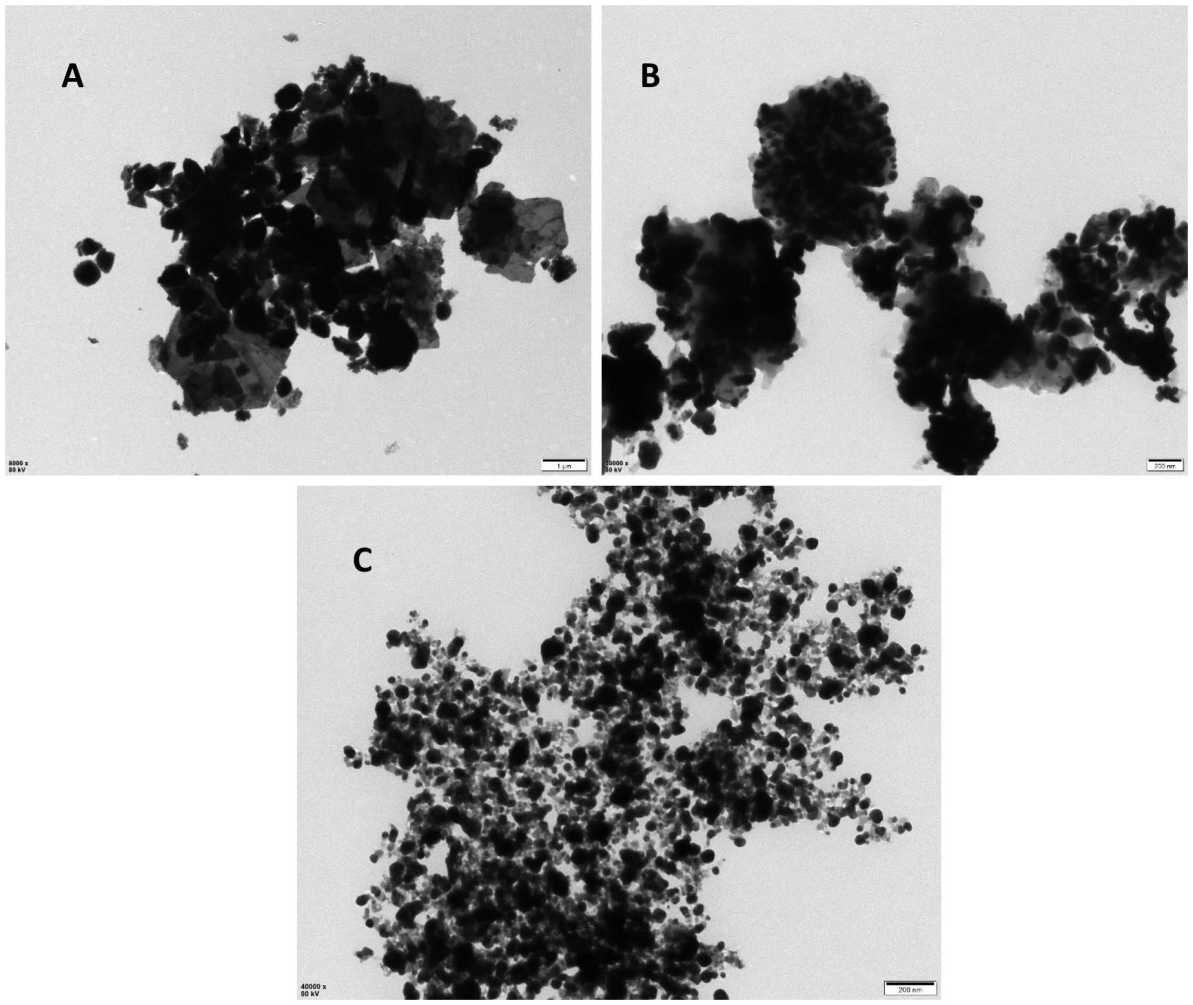
TEM images for the **(A)** CH, **(B)** CuO, and **(C)** CH-CuO nanoparticles.

The results of the UV–vis analysis are presented in Figure 1, which shows the different absorption ranges for the nanoparticles. The spectra for the CuO, CH, and CH-CuO nanoparticles exhibited absorption peaks at 255 nm, 300 nm, and 275 nm, respectively, which indicates the formation of nanoparticles. The SEM and TEM results for the analysis of the surface morphology of the nanoparticles are presented in Figures 2, 3, respectively. The SEM analysis showed a homogenous distribution of the nanoparticles present in the form of agglomerates, while the images of TEM depicted a spherical to roughly spherical shape of the nanoparticles. The histogram developed from the SEM results indicated an average particle size of 62 nm, 45 nm, and 51 nm for the CH (Figure 2A), CuO (Figure 2B), and CH-CuO nanoparticles (Figure 2C), respectively.

**Figure 4:**
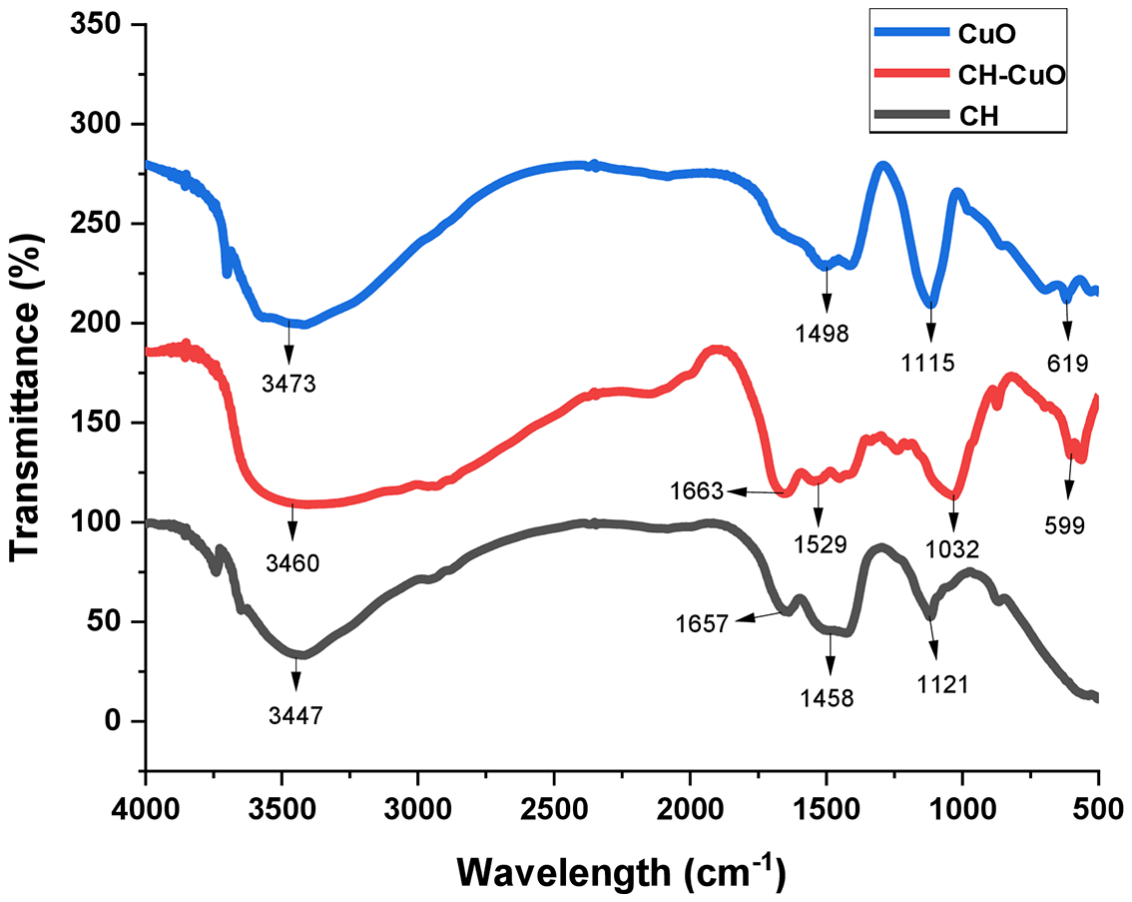
FTIR spectra depicting presence of various functional groups for the bio-synthesized CH, CuO, and CH-CuO nanoparticles.

The FTIR analysis for all the nanoparticles showing absorption bands at various ranges is presented in Figure 4. The results indicated that the CH, CuO, and CH-CuO spectra showed a broad absorption band at 3447 cm^−1^, 3,473 cm^−1^, and 3,460 cm^−1^, respectively. This broad peak in each spectrum indicates the presence of -OH groups, which can be attributed to the plant extract (polyphenols) that was used for the synthesis of the nanoparticles. Furthermore, chitosan also contributes to the -OH broad band due to the presence of primary and secondary -OH in its structure. The absorption bands at 1657 cm^−1^ and 1,663 cm^−1^ obtained for the CH and CH-CuO nanoparticles, respectively, correspond to presence of the amide group of chitosan; the characteristic peak was absent in the CuO nanoparticles. Peaks at 1458 cm^−1^, 1,498 cm^−1^, and 1,516 cm^−1^ showed the presence of C-H, while peaks at 1121 cm^−1^, 1,115 cm^−1^, and 1,032 cm^−1^ represented C-H stretching. Sharp peaks were obtained at 619 cm^−1^ and 599 cm^−1^ for the CuO and CH-CuO nanoparticles, respectively, which represented the presence of the Cu-O bond; the peak was absent in the case of CH nanoparticles.

**Figure 5:**
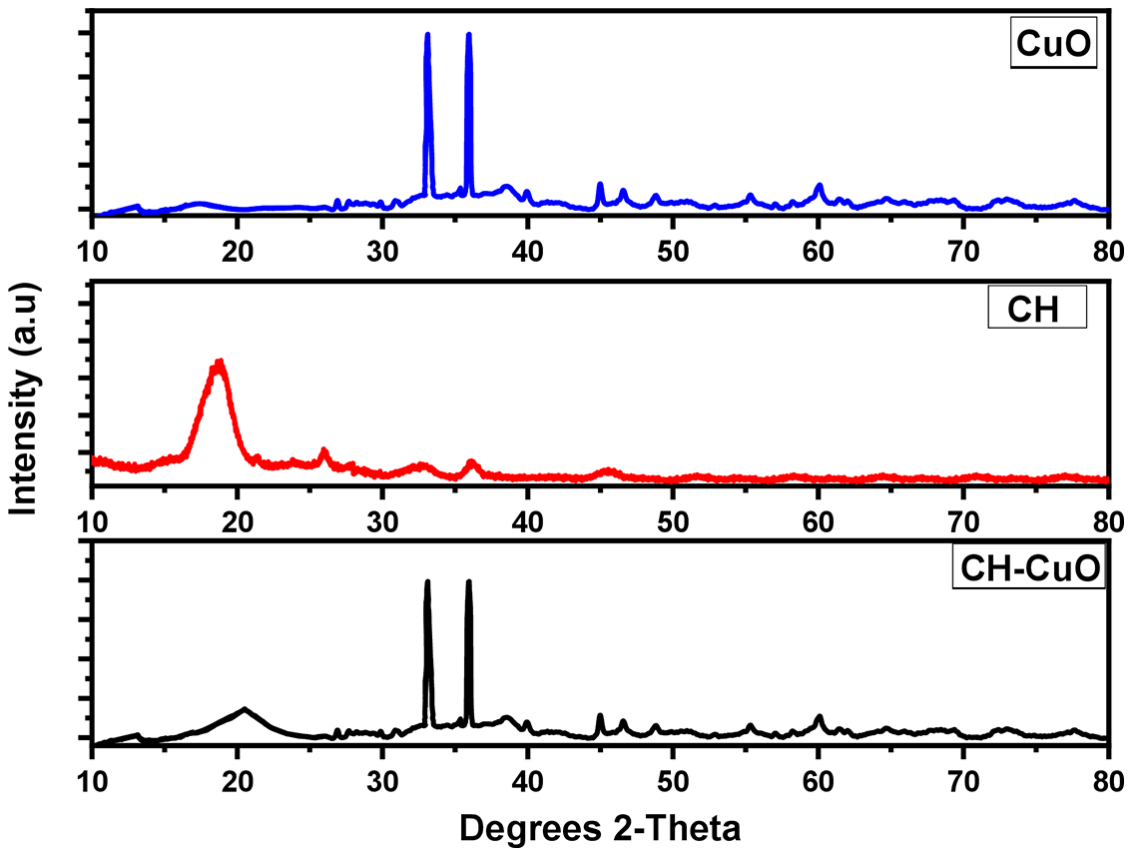
XRD pattern for the CH, CuO, and CH-CuO nanoparticles.

The XRD pattern for the nanoparticles is presented in Figure 5. The diffraction showed sharp peaks for the CuO and CH-CuO nanoparticles, which proved the crystalline structure of the nanoparticles. For CH, the obtained pattern showed comparatively broader peaks, which indicated slight amorphicity for these nanoparticles. Broader peaks at 18.64° and 20.12° for the CH and CH-CuO nanoparticles, respectively, indicated the presence of chitosan; the peaks were not observed for the CuO nanoparticles. Meanwhile, two long and sharp peaks at 33.03° and 35.96° were obtained for both the CuO and CH-CuO nanoparticles, respectively, which, in accordance with previous literature, corresponds to the monoclinic phase of CuO. These sharp peaks were not observed in the XRD spectrum of the CH nanoparticles. The average crystallite size was calculated for the nanoparticles using the Debye–Scherrer equation as follows:


d=Kλ/βcosθ

where *d* represents the crystallite size (nm), K represents the Scherrer constant (0.9), 𝜆 represents the wavelength (0.15406), *β* is the full width at half maximum of the respective peaks (FWHM), and *θ* represents the peaks positions (radians) from the graph. The average crystallite size for the CH, CuO, and CH-CuO nanoparticles was 33.54 nm, 20.13 nm, and 24.14 nm, respectively.

### 3.2. Antibacterial activity

**Figure 6:**
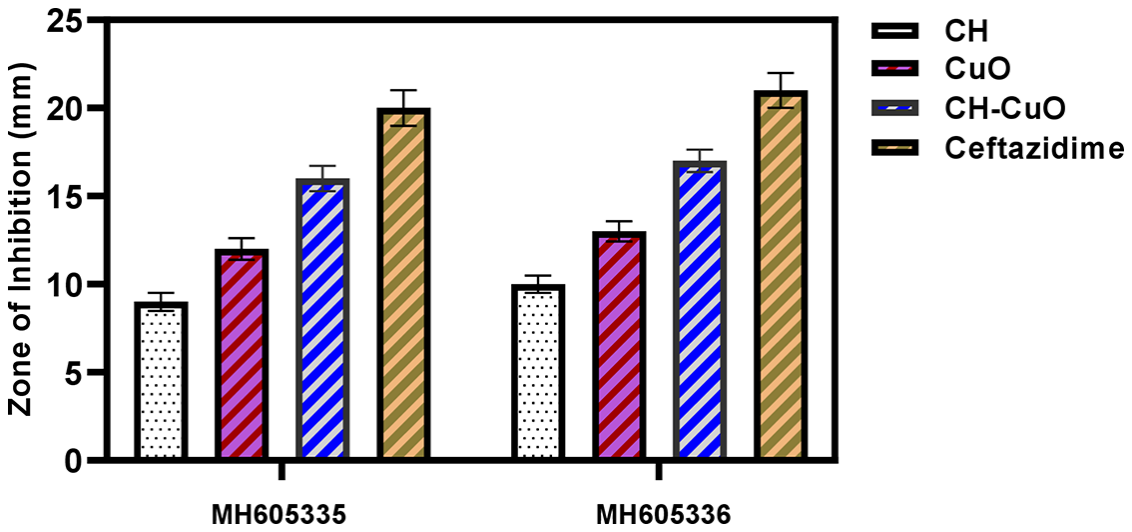
Antibacterial activity of the nanoparticles against *A. baumannii* isolates.

The susceptibility of *Acinetobacter baumannii* isolates was evaluated by an agar diffusion assay and MIC and MBC determination. The results for the antibacterial activity of the nanoparticles determined by the agar well diffusion assay are presented in Table 1. The activity of the nanoparticles was similar against both the bacterial isolates. The results in terms of the zone of inhibition show that all the nanoparticles displayed significant activity against *Acinetobacter baumannii* isolates (Figure 6). Maximum zones were obtained for the CH-CuO nanoparticles, which showed inhibition zones of 17 nm and 16 nm for MH605336 and MH605335, while the CuO nanoparticles showed comparatively moderate inhibitions of 13 nm and 12 nm, respectively. The chitosan nanoparticles were also found to possess antibacterial potential; however, their activity was least in comparison to the CuO and CH-CuO nanoparticles, as shown in Table 1.

**Table 1:** Zone of inhibition obtained for the nanoparticles against *Acinetobacter baumannii* isolates.

Bacterial isolate	Diameter of zone of inhibition (nm)			
	CH	CuO	CH-CuO	Ceftazidime
A. *baumannii* MH605335	9 ± 0.52	13 ± 0.61	17 ± 0.72	21 ± 1.02
A. *baumannii* MH605336	10 ± 0.49	12 ± 0.58	16 ± 0.64	20 ± 0.98

MIC and MBC were evaluated through a broth microdilution assay by exposing the bacterial isolate against different concentrations of nanoparticles. The results for the inhibitory and bactericidal concentrations for the nanoparticles showed a similar trend for the bacterial isolates, as shown in Table 2. The best results were obtained for CH-CuO, where the least values were exhibited by the nanoparticles. The CH and CuO nanoparticles displayed MIC and MBC values of 125 μg/mL and 500 μg/mL for both the bacterial isolates, respectively. Meanwhile, for CH-CuO, 62.55 μg/mL and 250 μg/mL were the minimum inhibitory and bactericidal concentrations against the bacterial isolates, respectively.

**Table 2:** MIC and MBC values for the nanoparticles against *Acinetobacter baumannii* isolates.

Bacterial isolate	MIC			MBC		
	CH	CuO	CH-CuO	CH	CuO	CH-CuO
A. *baumannii* MH605335	125 ± 5.42	125 ± 4.72	62.5 ± 4.51	500 ± 13.21	500 ± 15.72	250 ± 7.59
A. *baumannii* MH605336	125 ± 6.02	125 ± 5.65	62.5 ± 3.42	500 ± 14.58	500 ± 13.64	250 ± 8.81

### 3.3. Antibiofilm activity

**Figure 7:**
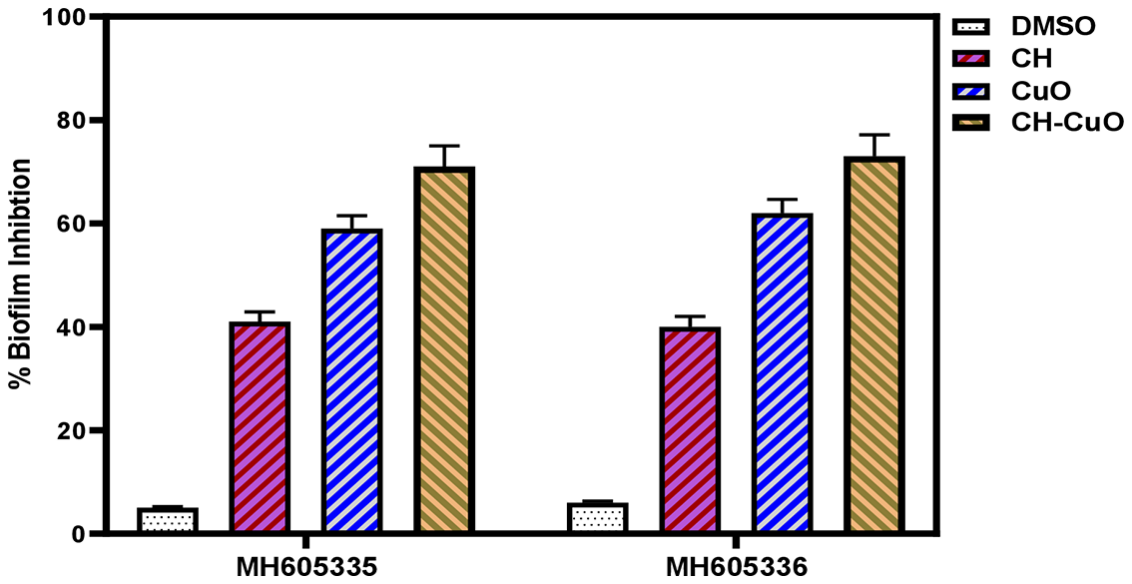
Biofilm inhibition activity for the CH, CuO, and CH-CuO nanoparticles against the bacterial isolates.

For determining the antibiofilm activity of the nanoparticles, the bacterial isolates ware incubated in the presence and absence of the nanoparticles, and a crystal violet staining assay was used to determine the biofilm inhibition. The results for the control and treated bacteria, presented in Figure 7, show effective biofilm inhibition activity for the nanoparticles. The antibiofilm activities for the nanoparticles were obtained in the order of CH-CuO > CuO > CH. For MH605335, biofilm inhibitions of 41, 59, and 71% were achieved by the CH, CuO, and CH-CuO nanoparticles, respectively, while 40, 62, and 73% antibiofilm activities were obtained against the MH605336 isolate. Therefore, the CH-CuO nanoparticles showed maximum antibiofilm potential against both isolates of *A. baumannii*.

### 3.4. Antioxidant activity

**Figure 8:**
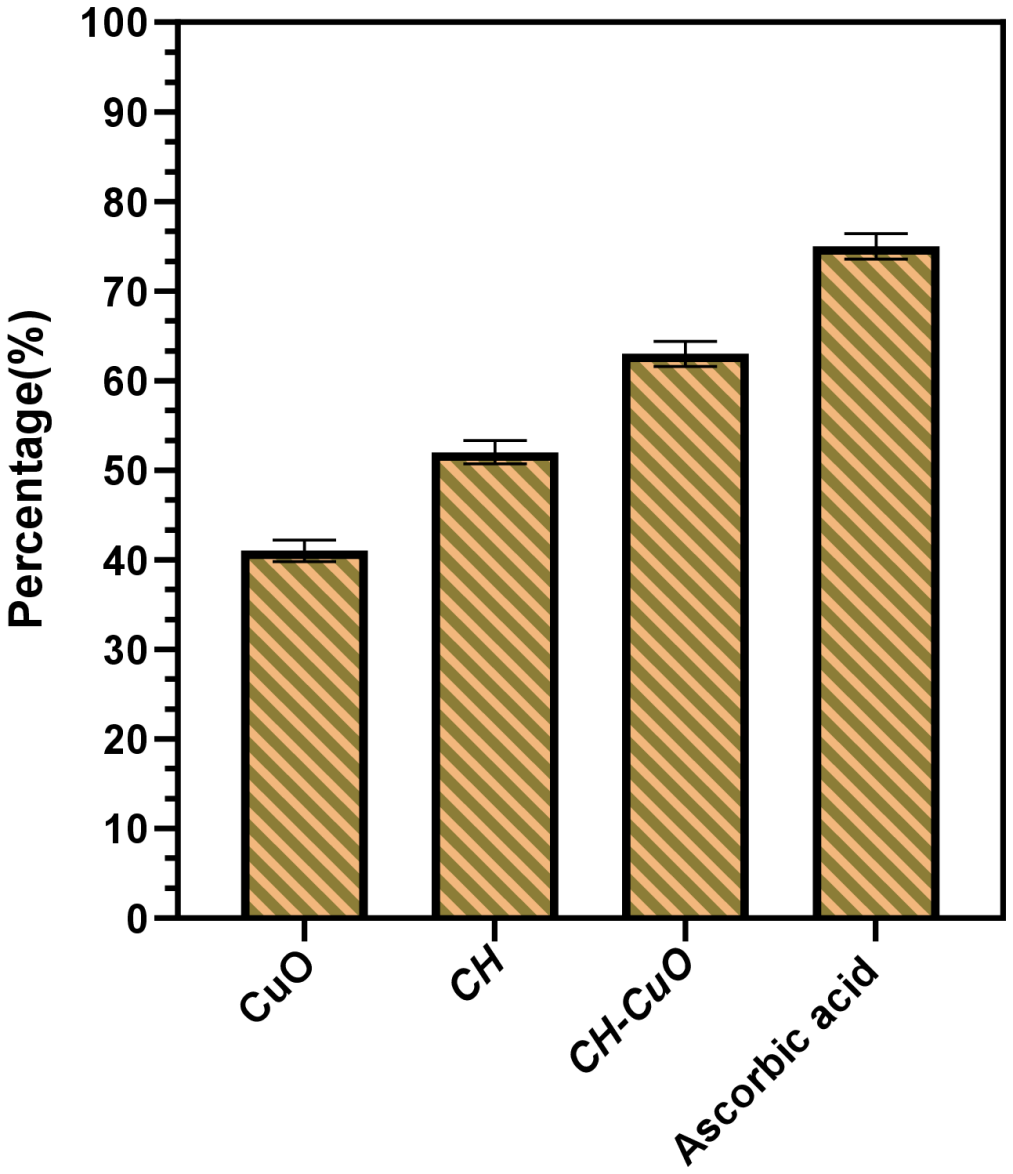
DPPH scavenging activity for the nanoparticles in comparison with the standard, ascorbic acid.

DPPH free radical scavenging activity was evaluated to determine the antioxidant potential of the nanoparticles. The results for the antioxidant activity are presented in Figure 8. The activities for the nanoparticles were compared with the standard, which showed maximum radical scavenging activity of 75%. In comparison to the standard, the antioxidant activity was comparatively less for the CH and CuO nanoparticles. CH-CuO presented comparatively better scavenging potential, where the activity of 63% was obtained, while the CH and CuO nanoparticles exhibited 52 and 41% activity, respectively.

### 3.5. Cytotoxicity and anticancer activity

**Figure 9:**
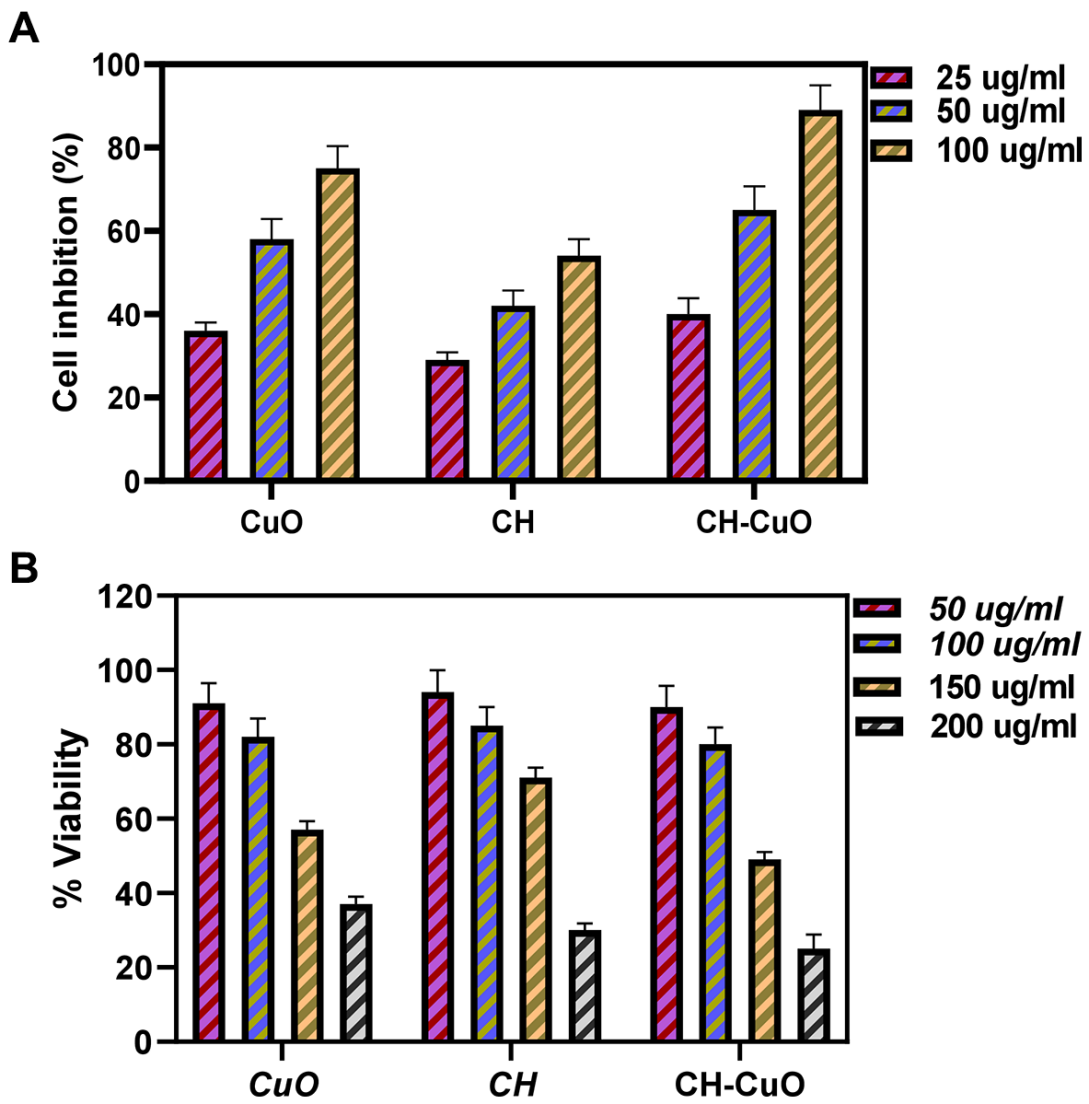
**(A)** Cytotoxicity analysis for the CH, CuO, and CH-CuO nanoparticles against Vero cell lines. **(B)** Anti-cancer activity for the CH, CuO, and CH-CuO nanoparticles against HepG2 cell lines at different concentrations.

*In vitro* cytotoxicity and anticancer activities were determined by an MTT assay against Vero cell lines and HepG2 cell lines, respectively. The results evaluated by assessing the cell viability and cell mortality are presented in Figures 9A,B, respectively. The nanoparticles exhibited a cytotoxic effect in a concentration- dependent manner, as the cell viability was decreased with the increase in the concentration of the nanoparticles. The results indicate that the cell lines show viability of 80% and above at 50 µg/ml and 100 µg/ml concentrations of the nanoparticles, which can be considered as an acceptable toxicity level. Cell viability (%) after exposure with 50 µg/ml of the CH, CuO, and CH-CuO nanoparticles was 93%, 89%, and 86%, respectively. At 100 µg/ml, the cell lines showed viability of 85%, 83%, and 80%, respectively, while at 150 ug/ml, the viability of the cells decreased to 72%, 59%, and 54%. SoTherefore, the concentration of the nanoparticles at 100 µg/ml can be deemed as acceptable for the normal cell lines.

**Figure 10:**
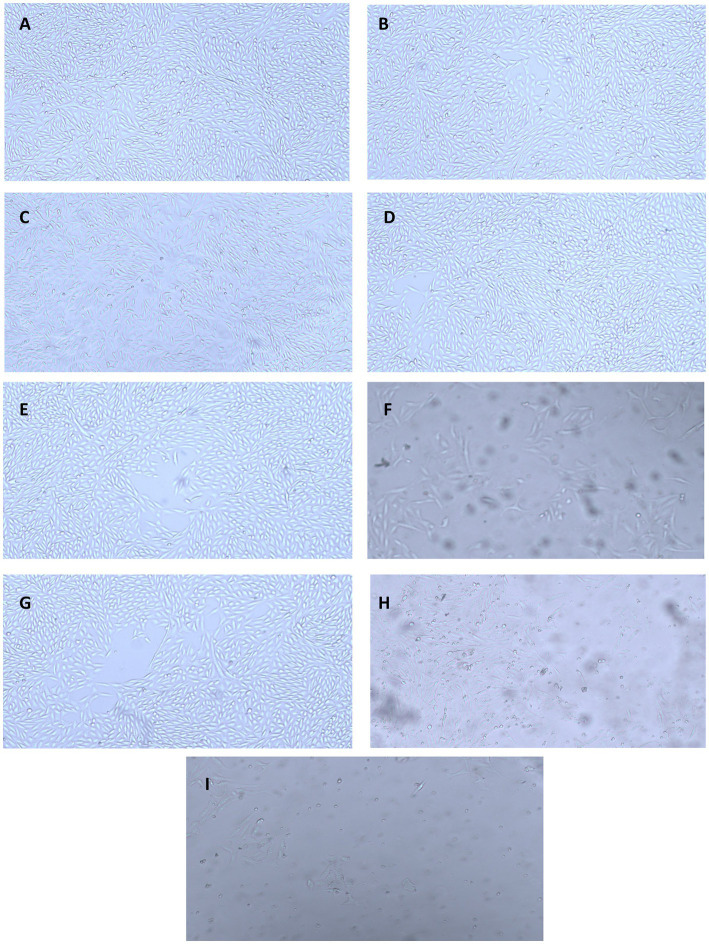
Microscopy images showing anti-proliferative activity of the nanoparticles against HepG2 cell lines at different concentrations: **(A)** CH (25 μg/mL), **(B)** CuO (25 μg/mL), **(C)** CH-CuO (25 μg/mL), **(D)** CH (50 μg/mL), **(E)** CuO (50 μg/mL), **(F)** CH-CuO (50 μg/mL), **(G)** CH (100 μg/mL), **(H)** CuO (100 μg/mL), and **(I)** CH-CuO (100 μg/mL).

The results for the anticancer activity evaluated by assessing the cell viability through the absorbance measurement are presented in Figure 9B. A decrease in the cell viability of HepG2 cell lines was observed owing to the cytotoxic effect of the nanoparticles. The nanoparticles exhibited anticancer activity in a concentration-dependent manner, where an increase in the activity was observed with the increase in the concentration. It was observed that the CH-CuO nanoparticles showed maximum suppression of cell growth, while the CH nanoparticles exhibited the least inhibition. Cell growth inhibition (%) after exposure with 100 μg/mL of nanoparticles for the CH, CuO, and CH-CuO nanoparticles was 54%, 75%, and 84%, respectively. The images for the microscopy of the cell lines showing morphological changes in the cells after exposure with the nanoparticles can be seen in Figure 10. The cells incubated with the nanoparticles underwentgo morphological changes from elongated epithelial to irregular rounded- shaped cells. As shown in the images, the HepG2 cells underwent cellular morphological changes, indicating unhealthy cell shrinkage. The deformities in the cell morphology are more visible at higher concentrations of nanoparticles, i.e., 50 μg/mL and 100 μg/mL. A visible decrease in the cell number can be seen for the CH-CuO nanoparticles, which was obtained at a 100 μg/mL concentration.

## 4. Discussion

The emerging antibiotic resistance is a major concern of the current era in which it is becoming difficult for antibiotics to cope with the pace of evolving drug-resistant pathogens. Furthermore, biofilm formation also provides an additional barrier to these pathogens, and the intractable infections caused by such biofilm-forming drug-resistant microbes are posing a great challenge for the healthcare sector (Abebe, 2020). Recently, nanotechnology has spanned over various multi-disciplinary areas and has been of interest in different fields of research. Nanoparticles have shown potential against various pathogens and are therefore of interest to researchers in dealing with the evolving resistance issue (El-Naggar et al., 2022). This study investigated the potential of biosynthesized nanoparticles for their antibacterial and antibiofilm activity against *Acinetobacter baumannii* isolates. The green-synthesized nanoparticles of CH, CuO, and CH-CuO were characterized by different techniques, where the SEM and TEM analyses showed roughly spherical-shaped nanoparticles with average sizes of 62 nm, 35 nm, and 51 nm, respectively. The calculated particle sizes for the nanoparticles were found to be within the range reported by the previous studies (Ali et al., 2011; Maheo et al., 2022).

The absorption spectrum of the nanoparticles was measured using UV–Vis spectrometry, which confirmed the synthesis of the nanoparticles (Gomathi et al., 2017). The nanoparticles possess specific optical properties depending upon the shape, size, and inter-particle distance; therefore, they respond to UV exposure by displaying a specific absorption spectrum (Khan et al., 2019). The study showed the absorption spectrum for the nanoparticles in which CuO displayed a sharp absorbance peak at 255 nm, while chitosan exhibited a shift in absorbance, and a broad peak was obtained at 300 nm. For the CH-CuO nanoparticles, it was found that the obtained spectrum was in between the CuO and CH nanoparticles, and the peak was obtained at 275 nm. The obtained results are in agreement with previous studies, including that of Dutta et al. (2015), who reported a peak at 258 nm for the CuO nanoparticles. Similarly, Rashki et al. (2022) reported the absorption peak for the chitosan nanoparticles to be around 300 nm. For the CH-CuO nanoparticles, Umoren et al. (2022) characterized the CH-CuO nanocomposites and reported the spectral absorbance peak for the nanocomposite at around 285 nm, while Maheo et al. (2022) reported the absorption peak for the green- synthesized CH-CuO nanoparticles at 278 nm.

The results of the FTIR analysis showed the presence of different functional groups at various ranges that indicated the presence of chitosan, CuO, and active phytochemicals of the plant extract in the nanoparticles. The absorptions range of each nanoparticles displayed a characteristic variation which that indicated that the distinct composition of each nanoparticle. The absence of the amide group in the CuO nanoparticles and the CuO group in the CH nanoparticles provided evidence for the successful synthesis of each characteristic nanoparticle. Javed et al. (2021) also provided a comparative analysis of the chitosan and CuO nanoparticles via FTIR, where in which they authors indicated a distinct absorption spectrum for each nanoparticle, i.e., CuO,. CH, and CH-CuO, which is in agreement with the current study. Similarly, other previous studies also reported the characterization of the CH and CH-CuO nanoparticles through an FTIR analysis and indicated the presence of various functional groups, as reported in this study (Nagajyothi et al., 2017; Logpriya et al., 2018). XRD analysis for the nanoparticles showed a crystalline nature for the nanoparticles where sharp and huge peaks were obtained for the CuO nanoparticles, indicating a perfect crystalline structure. For CH, it was observed that the peaks obtained were broader and less sharp, which indicated reduced the crystallinity of the chitosan nanoparticles. In the case of CH-CuO, it was observed that the intensity of the crystalline structure was slightly decreased by the addition of chitosan in the CuO nanoparticles. Similar findings have also been reported in pPrevious literature, such as has also reported similar findings where Maheo (2022), who observed sharp peaks for the metal oxide nanoparticles, while the crystalline nature was reduced for the CH-CuO nanoparticles. Similarly, Prasasti et al. (2018) also reported a decrease in the crystalline nature of the nanostructures by the addition of chitosan.

The antibacterial and antibiofilm potential was tested for each nanoparticle against *Acinetobacter baumannii* isolates. The nanoparticles effectively inhibited the growth of drug-resistant pathogens. The inhibitions were obtained in the order of CH-CuO > CuO > CH nanoparticles for both the strains. The antibacterial activity of the CuO nanoparticles against various drug-resistant pathogens has been reported in different studies (Azam et al., 2012a,b; Dadi et al., 2019). Javed et al. (2021) studied the comparative effect of the CH, CuO, and CH-CuO nanoparticles against various drug-resistant pathogens, and the best activity was reported for the CH-CuO nanoparticles. Umoren et al. (2022) reported the antimicrobial activity of olive leaf extract- mediated CH-CuO nanoparticles and highlighted effective activities against Ggram-negative (Pseudomonas aeruginosa and Escherichia coli) and Ggram-positive (Staphylococcus haemolytica and Bacillus cereus) bacterial species. Similarly, Wahid and co-workers (Wahid et al. (2017) used carboxymethyl chitosan/CuO nanocomposites and highlighted the antimicrobial potential against S. aureus and E. coli. Likewise, Paul et al. (2022) also reported the antibacterial potential for chitosan- coated CuO nanomaterial against both Ggram-positive and -negative bacteria.

The mechanism for the activity of the nanoparticles is based on the nanosize of the particles that attach with membranes of the microbes. The positively charged metal oxide nanoparticles, in this case CuO, bind with the lipophilic membranes due to the electrostatic interaction with the negatively charged cell membranes, hence affecting the membrane permeability of the cell. Furthermore, the nanosized nanoparticles can also enter the cell and attach with DNA and proteins to inhibit the cellular processes. The metal oxides can also form reactive species, which are highly reactive and can damage membranes and internal cellular components (Prabhu and Poulose, 2012; Dizaj et al., 2014; Meghana et al., 2015). The antimicrobial potential for both chitosan and chitosan nanoparticles has also been reported in different studies (Abdeltwab et al., 2019; Saberpour et al., 2020; Al-Zahrani et al., 2021). Chitosan imparts its antimicrobial effect owing to the presence of positively charged amino groups that can bind with the negatively charged microbial membranes inhibiting cell growth (Fang et al., 2020). In the current study, maximum inhibition was exhibited by the CH-CuO nanoparticles, as they consisted of both chitosan and CuO, which provided the combined antimicrobial effect.

Biofilm is a consortium of microorganisms in which the microbes live in association with each other in order to withstand external stress. Microbial cells in the biofilm are embedded in the matrix composed of extracellular polymeric substances (EPS). Biofilm provides an external barrier, which makes it difficult for antibiotics to penetrate the matrix, thus helping the pathogens to gain resistance against multiple antibiotics. The biofilm-forming pathogens can colonize medical devices and are responsible for causing many calcitrant infections in healthcare settings (Gedefie et al., 2021). The current study showed effective biofilm inhibition by the nanoparticles against *Acinetobacter baumannii* strains, with the best activity obtained for the CH-CuO nanoparticles. Previous literature is also in agreement with these results, in which the antibiofilm potential has also been reported for the CH, CH-CuO, and CuO nanoparticles against different drug-resistant strains (Ansarifard et al., 2021; Cai et al., 2022; Umoren et al., 2022). Agarwala et al. (2014) reported successful biofilm inhibitions for *E. coli* and *Staphylococcus aureus* by employing the CuO nanoparticles, while Logpriya et al. (2018) synthesized the CH-CuO nanocomposites and reported 69 % and 63 % biofilm inhibition against *Bacillus subtilis* and *Pseudomonas aeruginosa*, respectively. Covarrubias et al. (2018) evaluated the potential of hybrid nanostructures, consisting ofcomprising the CuO nanoparticles with a chitosan shell, for eradicating dental biofilms, and they reported effective activity against Streptococcus mutans biofilms. Similarly, Pérez-Díaz et al. (2016) used chitosan with silver nanoparticles and reported effective antibiofilm activity by exhibiting a log -reduction of 6.0 for MRSA.

Maximum inhibition was achieved by the CH-CuO nanoparticles against both strains. The mechanism for this activity has been attributed to the positively charged nature of chitosan. The biofilms are generally negatively charged, which is imparted majorly by the external matrix (EPS), while the chitosan structure has N-acetylglucosamine subunits that contain positively charged amino groups (NH3+). Thus, the electrostatic attraction between the oppositely charged entities results in the interaction between the nanoparticles and biofilm matrix, hence affecting the flow of materials across the matrix. The nanoparticles can also penetrate the biofilms, where they can affect the quorum-sensing ability to inhibit biofilm development (Divya et al., 2017; Shkodenko et al., 2020). The particle size is also one of the important factors for the antibiofilm activity of the CH-CuO nanoparticles. The smaller particles possess a larger surface area for interacting with the bacterial surfaces, i.e., cell wall and cell membrane, which limits bacterial growth. Furthermore, CH-CuO releases Cu2+ ions which that covers the bacterial surface and alter thes structure of the surface proteins, which thus affectings the attachment of the cells on the substratum. Furthermore, they enter the cells and interact with the DNA and enzymes inside the cells, which affects biofilm and cell development (Das et al., 2021).

Antioxidant activity evaluation is one of the most actively performed assessment parameters for nanoparticles. The activity of antioxidants has huge implications on the bodies where the free radicals, which potentially play a role in cancer and heart diseases, are neutralized by the radical scavenging agents (Dobrucka, 2018). In this study, the antioxidant potential for the nanoparticles was determined by the DPPH radical scavenging activity, where the nanoparticles exhibited effective activity, and the best results were obtained for the CH-CuO nanoparticles. Effective antioxidant activities for the CH, CuO and CH-CuO nanoparticles have also been reported in previous literature (Divya et al., 2018; Singh et al., 2018; Javed et al., 2021). The mechanism for the antioxidant activity of the nanoparticles can be due to different possible reasons. A major contribution can be from the plant extract that was used for the synthesis of the nanoparticles, as the secondary metabolites from the extract (flavonoids, phenolic compounds, tannins) can get incorporated into the nanoparticles, which can contribute to the activity. Rehana et al. (2017) compared the activities of green and chemically synthesized CuO nanoparticles for their antioxidant properties and reported better activity for the particles synthesized via the biological route. Furthermore, chitosan possesses amino groups in a structure that can scavenge free radicals by donating hydrogen ion (Ivanova and Yaneva, 2020). Therefore, the combined action of the green synthesis and chitosan effectively contributed to the antioxidant activity for the CH-CuO nanoparticles.

Anticancer activity was evaluated via an MTT assay, in which the nanoparticles inhibited the HepG2 cell lines, and maximum inhibition was obtained for the CH-CuO nanoparticles. Previous studies have reported anticancer activity for the CuO nanoparticles (Rajamma et al., 2020; Shwetha et al., 2021). Similarly, the anticancer potential of chitosan has also been affirmed in some findings (Almutairi et al., 2020; Tuorkey et al., 2022). The findings of this study are in accordance with those of Javed et al. (2021), who compared the cytotoxic potential of the CH, CuO, and CH-CuO nanoparticles and reported best activity for the CH-CuO nanoparticles. The activity of the CH-CuO nanoparticles can be attributed to the combined effect of chitosan and CuO. Adhikari and Yadav (2018) suggested that chitosan possesses positively charged amino groups, while the cell membrane of the cancer cells is highly negative in comparison to the normal cells. Thus, chitosan is attracted to the cancer cells and attacks the cells *via* membrane interaction, due to electrostatic interactions between the opposite charges, or through endocytosis extracellularly, which results in inhibiting the cell proliferation.

## 5. Conclusion

This study discussed a comparative analysis of the CH, CuO, and CH-CuO nanoparticles, synthesized via the green approach, to determine their potential in the healthcare sector. The nanoparticles were evaluated for their antibacterial, antibiofilm, and anticancer applications. The nanoparticles displayed effective activities against MDR *Acinetobacter baumannii* strains and inhibited biofilm formation, which can have huge implications on dealing with hospital infections. The bioassays for antioxidant and anticancer activities also confirmed potent activities for all the nanoparticles. In terms of comparison, the study reported best results for the CH-CuO nanoparticles for all the bioassays due to the combined effect of chitosan and CuO. The findings of this study suggest that the CH, CuO, and CH-CuO nanoparticles have potential applications in the healthcare sector, while the comparative analysis concludes that the CH-CuO nanoparticles could be the most promising candidate for dealing with multi-drug resistance infections and cancers. However, further *in vivo* investigations are suggested to unfold the in-depth potential activities and cytotoxicity of the nanoparticles in practical settings along with the challenges surrounding their large-scale commercial production.

## Data availability statement

The raw data supporting the conclusions of this article will be made available by the authors, without undue reservation.

## Author contributions

Conceptualization, MS, SM; methodology, MS, MZ; Material preparation, MS, BA, and MS; data determination and statistical analysis, MS, AA and SH; writing—original draft preparation, MS and MK; writing-review and editing, AH, TD, GA-Q, EA and JC; supervision, SM. All authors have read and agreed to the published version of the manuscript.

## Funding

The authors would like to extend their sincere appreciation to the Researchers Supporting Project, number (RSP2023R134), King Saud University, Riyadh, Saudi Arabia.

## Conflict of interest

The authors declare that the research was conducted in the absence of any commercial or financial relationships that could be construed as a potential conflict of interest.

## Publisher’s note

All claims expressed in this article are solely those of the authors and do not necessarily represent those of their affiliated organizations, or those of the publisher, the editors and the reviewers. Any product that may be evaluated in this article, or claim that may be made by its manufacturer, is not guaranteed or endorsed by the publisher.
